# The Risk of Achilles Tendon Rupture in the Patients with Achilles Tendinopathy: Healthcare Database Analysis in the United States

**DOI:** 10.1155/2017/7021862

**Published:** 2017-04-30

**Authors:** Youichi Yasui, Ichiro Tonogai, Andrew J. Rosenbaum, Yoshiharu Shimozono, Hirotaka Kawano, John G. Kennedy

**Affiliations:** ^1^Department of Orthopedic Surgery, Teikyo University School of Medicine, Tokyo, Japan; ^2^Hospital for Special Surgery, New York, NY, USA; ^3^Department of Orthopedic Surgery, Tokushima University, Tokushima, Japan; ^4^Albany Medical Center, Albany, NY, USA

## Abstract

*Introduction*. Disorders of the Achilles tendon can be broadly classified into acute and chronic entities. Few studies have established chronic Achilles tendinopathy as a precursor to acute Achilles ruptures. In this study, we assessed the relationship between Achilles tendinopathy and rupture, clarifying the incidence of rupture in the setting of underlying tendinopathy.* Methods*. The United Healthcare Orthopedic Dataset from the PearlDiver Patient Record Database was used to identify patients with ICD-9 codes for Achilles rupture and/or Achilles tendinopathy. The number of patients with acute rupture, chronic tendinopathy, and rupture following a prior diagnosis of tendinopathy was assessed.* Results. *Four percent of patients with an underlying diagnosis of Achilles tendinopathy went on to sustain a rupture (7,232 patients). Older patients with tendinopathy were most vulnerable to subsequent rupture.* Conclusions*. The current study demonstrates that 4.0% of patients who were previously diagnosed with Achilles tendinopathy sustained an Achilles tendon rupture. Additionally, older patients with Achilles tendinopathy were most vulnerable. These findings are important as they can help clinicians more objectively council patients with Achilles tendinopathy.

## 1. Introduction

Achilles tendon disorders are commonly encountered in both the athletic and general populations [1]. Disorders can be divided into two general categories: acute and more chronic overuse injuries. Acute rupture of the Achilles tendon most frequently occurs in males between 30 and 40 years of age [2]. Although it is considered an acute process, histological analyses have demonstrated that, even in the setting of acute rupture, degenerative changes are regularly found within the tendon [3–8]. Achilles tendinopathy is a more indolent and chronic process and is attributed to repetitive overuse. It is most prevalent in individuals aged 20–60 years [9]. In the setting of Achilles tendinosis, histological analysis reveals degenerative changes within the tendon [10, 11].

The histological similarities between acute Achilles tendon ruptures and chronic tendinopathy suggest that some individuals may sustain a presumed acute rupture in the setting of the more chronic tendinopathy. However, little is known about this, with studies presenting conflicting findings. While some works suggest that rupture following tendinopathy occurs in 5% to 44% of individuals [12, 13], others have shown that most patients with tendinopathy have favorable functional outcomes without tendon rupture [14–16].

Through the use of a deidentified patient database, this study clarifies the risk of Achilles tendon rupture in patients with a formal diagnosis of Achilles tendinopathy.

## 2. Methods

### 2.1. Data Source

Data was obtained from the United Healthcare Orthopedic (UHC) dataset from the PearlDiver Patient Record Database (PearlDiver Technologies, Inc., Fort Wayne, IN, USA). This database is comprised of deidentified patients in a Health Insurance Portability and Accountability Act (HIPAA) compliant fashion [17, 18].

The UHC database consists of reported data from hospitals and/or physicians between 2007 and 2011 and has information on 20,484,172 patients. Approximately 9% of the United States (US) population younger than 65 years of age and approximately 13% of the US population with private medical insurance are represented in the database [19]. Additionally, the PearlDiver Patient Record Database includes all patients who enrolled with the insurance carrier during the desired time period before or after a specified event [17].

In the database, International Classification of Disease, Nine Codes (ICD-9), or current procedural terminology (CPT) codes are used to search for subsets of patients. Demographic information, such as age and gender, can then be assessed for these patients.

### 2.2. Cohort Selection

Three subsets of patients, all between 20 and 69 years old, were evaluated in this study: Group 1: patients with an acute Achilles tendon rupture; Group 2: patients with Achilles tendinopathy; and Group 3: patients with an Achilles tendon rupture following a diagnosis of Achilles tendinopathy.

The ICD-9 codes used in this study were 72767 (Achilles tendon rupture) and 72671 (Achilles tendinopathy). The number of patients was determined for each of the three cohorts. The incidence of each condition per 100,000 patients was calculated by dividing the number of patients with each disorder by the total number of patients aged 20–69 years in the UHC database (20,086,126 patients). The relationship between the disorders and demographic factors was assessed. The incidence of Achilles tendon rupture in the setting of Achilles tendinopathy was calculated by dividing the total number of patients with an Achilles tendon rupture following a diagnosis of Achilles tendinopathy (Group 3) by the total number of patients with Achilles tendinopathy (Group 2). Additionally, in each age group, the number of patients with an Achilles tendon rupture following a diagnosis of Achilles tendinopathy (Group 3) was divided the number of patients with Achilles tendinopathy (Group 2).

### 2.3. Statistical Analysis

Statistical analysis was performed using SAS 9.3 (SAS Institute, Cary, NC). Analysis of variance and Tukey's test were used to assess the incidence in each age and the chi-square test was applied for gender analysis. A *p* value of less than 0.05 was considered a statistically significant outcome.

## 3. Results

### 3.1. Group 1: Patients with an Acute Achilles Tendon Rupture

**Figure 1:**
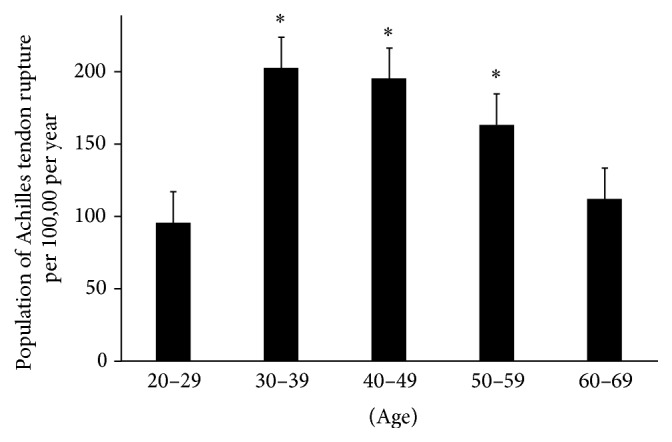
Group 1: population distribution in each age according to incidence of Achilles tendon rupture. ^∗^Most affected age groups.

A total of 21,305 patients (106 per 100,000) were included. Individuals aged 30–39 years were most often affected, followed by those aged 40–49 years. The incidence of Achilles tendon rupture in these age groups was significantly higher than that observed in individuals aged 20–29 years and 60–69 years (*p* < 0.05) (Figure 1). Males sustained ruptures more frequently than females (male versus female; 141 per 100,000 versus 67 per 100,000, resp.; *p* < 0.05).

### 3.2. Group 2: Patients with Achilles Tendinopathy

**Figure 2:**
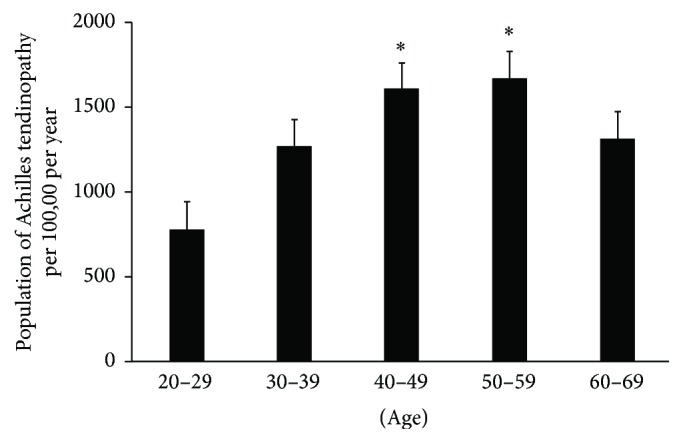
Group 2: population distribution in each age according to incidence of Achilles tendinopathy. ^∗^Most affected age groups.

A total of 180,421 patients (898 per 100,000) were diagnosed with Achilles tendinopathy. Individuals aged 50–59 years were most often affected, followed by those aged 40–49 years. The incidence of Achilles tendinopathy in those groups was significantly higher than that seen in those aged 20–39 years and 60–69 years (*p* < 0.05) (Figure 2). There were no statistical differences in the incidence of Achilles tendinopathy between males and females (male versus female; 892 per 100,000 versus 840 per 100,000, resp.;* n.s.*).

### 3.3. Group 3: Patients with an Achilles Tendon Rupture following a Diagnosis of Achilles Tendinopathy

**Figure 3:**
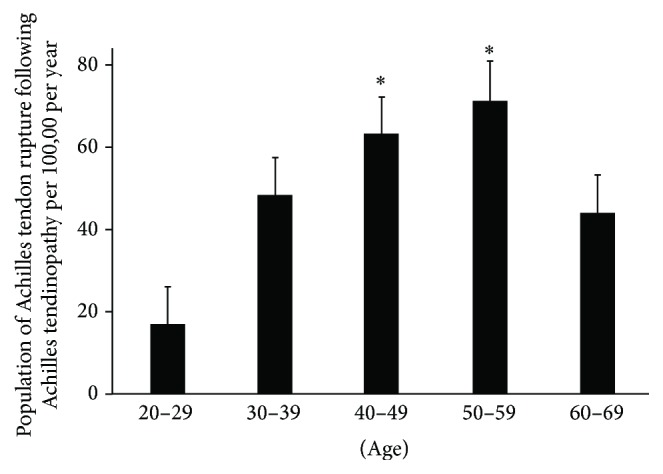
Group 3: population distribution in each age according to incidence of Achilles tendon rupture following Achilles tendinopathy. ^∗^Most affected age groups.

There were 7,232 patients (36 per 100,000) who sustained an Achilles tendon rupture following a diagnosis and treatment for Achilles tendinopathy (Figure 3). Those aged 50–59 years were most often affected, followed by those aged 40–49 years (4.3% and 3.9%, resp.). The incidence in these groups was significantly higher than that observed in those aged 20–39 and 60–69 years (*p* < 0.05) (Figure 3). There was no significant difference in the incidence of Achilles tendinopathy between males and females (male versus female; 41 per 100,000 versus 29 per 100,000, resp.).

### 3.4. The Relationship between Achilles Tendon Rupture and Achilles Tendinopathy

**Figure 4:**
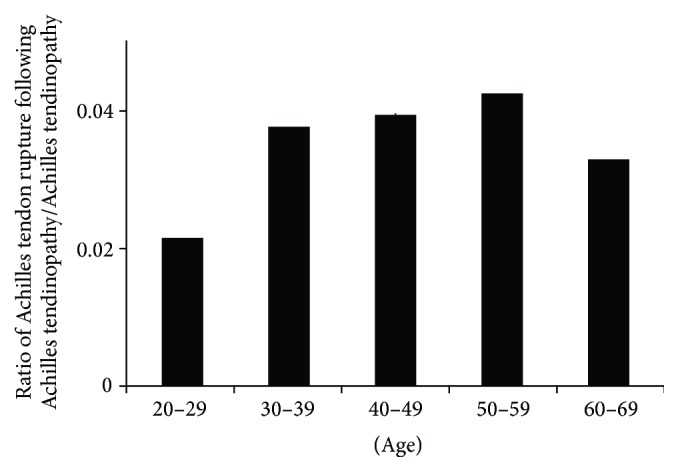
Distribution in each age according to ratio of incidence of Achilles tendon rupture following Achilles tendinopathy divided by incidence of Achilles tendinopathy.

Approximately 4.0% of patients with Achilles tendinopathy subsequently sustained a rupture (Figure 4). Individuals aged 50–59 years were most susceptible (4.3% incidence).

Admittedly, the time point between the diagnosis of tendinopathy and subsequent rupture was not ascertainable from current database.

## 4. Discussion

The present study analyzed a large, diverse population of individuals aged 20–69 years in order to determine the rate of Achilles tendon rupture in the setting of underlying Achilles tendinopathy. We found that approximately 4.0% of patients who were previously diagnosed with Achilles tendinopathy ultimately sustained a rupture. Additionally, older patients with tendinopathy were most vulnerable.

Intrasubstance degeneration of the Achilles tendon has been found in individuals with both acute rupture and chronic tendinopathy [3–8, 10, 11], suggesting that tendinopathy precedes and may even predispose individuals to Achilles tendon rupture [20]. However, studies have reported inconsistent outcomes. Maffulli found that 5% (9/176) of patients who sustained an Achilles tendon rupture had previous symptoms over their Achilles tendon [12]. In a work by Nestorson et al. [13], 44% of patients (11/25) had Achilles tendon pain before Achilles tendon rupture. These studies must be interpreted with caution as they are comprised of small cohorts with uncontrolled variables.

We found that 4.0% of patients previously diagnosed with Achilles tendinopathy suffered an Achilles tendon rupture. This is an important finding and elucidates the intimate, but complex, relationship between Achilles tendinopathy and rupture. The small percentage of patients who went on to rupture following a diagnosis of tendinopathy (4.0%) underscores the success of the various treatment modalities specific to Achilles tendinopathy (e.g., eccentric stretching).

Outcomes from the current study suggest that age may be a risk factor for Achilles tendon rupture previously diagnosed with Achilles tendinopathy. Older patients with Achilles tendinopathy had a significantly higher risk of rupture than younger individuals. This finding is supported by a recent animal study that demonstrates the relationship between advancing age and degeneration of the Achilles tendon [21].

In this study, males had a higher risk of rupture. This too is consistent with other works, such as that by Wong et al. [22]. In that study, males were found to be 4–7 times more likely to rupture their Achilles tendon [22]. Although the reasons for this finding are currently unclear, this finding should be explored in future studies.

## 5. Limitations

As a database study, this work has inherent limitations. Pertinent patient information, such as injury mechanism, symptom severity, duration of symptoms, medical comorbidities, the degree of tendon degeneration, use of local and systemic corticosteroids, and fluoroquinolone usage, was unavailable. Although we found that 4.0% of patients with tendinopathy go on to rupture, our data can be presented differently, as it also suggests that 33.4% of patients who sustained a rupture were previously diagnosed with Achilles tendinopathy (Group 3/Group 1). In other words, 66.6% of patients who sustained an Achilles tendon rupture were not previously diagnosed with Achilles tendinopathy. While this can be interpreted as suggesting that Achilles tendon rupture is more common in the absence of tendinopathy, we do not believe that this is accurate. It is likely that some rupture patients may have had undiagnosed tendinopathy. Furthermore, we fail to account for the mechanism of injury in our analysis. In other words, patients rupturing in the absence of a tendinosis diagnosis may have been more likely to do so because of their given activities [17]. As previously mentioned, the database does not provide this information. The time between each diagnosis was also unknown. Another potential source of error is the possibility of any documentation or coding mistakes. We also do not know how tendinopathy patients were treated. This is important, as certain modalities (e.g., steroid injection) may have predisposed patients to rupture. Despite these limitations, we believe that the results from our large cohort of patients provide valuable insight into the relationship between Achilles tendinopathy and rupture.

## 6. Conclusions 

In this large cohort database study, we found that approximately 4.0% of patients who were previously diagnosed with Achilles tendinopathy sustained an Achilles tendon rupture. Additionally, older patients with Achilles tendinopathy were most susceptible to rupture. These findings are important as they can help clinicians more objectively council patients following a diagnosis of Achilles tendinopathy.
